# Book Review: *Pre and Probiotics for Poultry Gut Health*; Masey O’Neill et al., Eds.; CABI: Bakeham Lane, Egham, Surrey, UK, 2023; ISBN: 978-1-80062-272-2 (hbk)

**DOI:** 10.3390/ani13213385

**Published:** 2023-10-31

**Authors:** Velmurugu Ravindran

**Affiliations:** Monogastric Research Centre, School of Agriculture and Environment, Massey University, Palmerston North 4442, New Zealand; v.ravindran@massey.ac.nz

The poultry industry is facing a future without antibiotic growth promoters, the most effective and successful feed additive available. The ban in some regions, especially in the European Union, and degrees of voluntary withdrawal in others, spurred by reports of potential antibiotic resistance in humans, are adding pressure on the gut health and general health of poultry. The use of antibiotic growth promoters in poultry feeding is a key consumer issue and this challenge provides novel opportunities in the search for antibiotic alternatives. In this context, this book, edited by H.M. O’Neill, E. Burton and D. Scholey, is a useful contribution. The book is based on papers presented at the 33rd Poultry Science Symposium held in Cambridge University in August 2022.

The book is organised into 13 chapters, each dealing with different aspects relating to antibiotic resistance and antibiotic alternatives. The title of the book (‘Pre and Probiotics for Poultry Gut Health’), however, is somewhat misleading and implies a specific focus on prebiotics and probiotics. This is not the case, and only two of the 13 chapters specifically deal with these two feed additives. The remaining chapters deal with various scientific and practical issues relating to antibiotic use that, considered together, provide up-to-date insights. The editors must be congratulated for compiling the state-of-the-art knowledge of this complex area.

The chapters follow a logical progression, providing coherent coverage of key science and technology aspects of in-feed antibiotic use. Antibiotic resistance is an inherent side effect of using the antibiotics and is driving their ban in poultry diets. Chapter 1 sets the scene by providing a good overview of this complex issue and the challenges faced in antimicrobial stewardship.

The modes of action of in-feed antibiotics in growth promotion are multi-factorial; however, they essentially work through the modulation of gut flora. Current knowledge of poultry gut flora and alternative means of manipulating flora to address the issues of gut health and antimicrobial resistance are discussed in Chapter 2.

Approaches to managing poultry production in the post-antibiotic era are discussed in the next two chapters. A simplified and useful discussion of practical options to be considered in a global approach of antibiotic stewardship at the farm level is provided in Chapter 3. A short review of diet formulation strategies under commercial conditions is presented in Chapter 4.

Chapter 5 discusses three aspects of interest in depth, namely, zoonotic pathogens, antimicrobial resistance and food safety in relation to pre-, pro- and synbiotics. In particular, the usefulness of these biotics, along with the utility of other therapies such as bacteriophages, in food safety is highlighted.

The use of competitive exclusion to manage and modulate gut flora has long been known but has received little attention as an alternative for the use of in-feed antibiotics. This subject is comprehensively discussed in Chapter 6.

The literature abounds with reports of various feed additives consistently and markedly changing the numbers and diversity of microbial species in the gut, but none have been able to demonstrate any association between these changes and the key commercial performance indicators of poultry. This pertinent, and important, aspect of study and the need for future research are highlighted in Chapter 7.

Feed additives are the most researched subject area to date in poultry nutrition. It is demanding to analyze and interpret the voluminous published data, and these task are complicated by the availability of a large number of commercial products within each class of additive. One often finds that the research conditions employed in additive evaluation are not fully disclosed. The pitfalls in data collection and data analysis in reviewing the literature on additives are elegantly discussed in Chapter 8. The potential use of meta-analysis and holo-analysis is compared in a clear and simple manner.

There is an interesting section on xylo-oligosaccharides, one of the end products of hydrolytic degradation of xylans by endo-xylanases. Recent data on the mechanisms by which this prebiotic benefits poultry performance are critically analyzed in Chapter 9.

A useful discussion of the historical perspectives and current status of the legal framework covering feed additives in the European Union is provided in Chapter 10. This legislations not only drives changes in the European Union, but also in the global food chain. The most recent paradigm shift from March 2021—the introduction of the European Union transparency legislation—is not widely known and is lucidly explained in this chapter.

Chapter 11 deals with the potential application of bacteriophage therapy to improve poultry health. The pros and cons of phage therapy are discussed. In Chapter 12, an overview of phototherapeutics, medicines derived from plants, is provided and, their practical use and the author’s field experience are highlighted. In the final chapter, recent published data on biomarkers for intestinal health are reviewed and future possibilities are explored.

Overall main, this book combines an array of topical issues relating to antibiotic alternatives and constitutes a valuable contribution to the ongoing debate. The bibliography, with citations up to 2021, is provided after each chapter for any reader wishing to delve further into a particular topic. This book provides useful reference material to researchers, university teachers, and students of poultry science. Those involved in the commercial industry will also benefit from the practical information provided.

